# The prevalence of malnutrition and its effects on the all-cause mortality among patients with heart failure: A systematic review and meta-analysis

**DOI:** 10.1371/journal.pone.0259300

**Published:** 2021-10-28

**Authors:** Shubin Lv, Songchao Ru

**Affiliations:** The First Affiliated Hospital, and College of Clinical Medicine of Henan University of Science and Technology, Luoyang, China; Sahlgrenska University Hospital, SWEDEN

## Abstract

**Objective:**

Malnutrition has a high occurrence in patients with chronic heart failure (CHF). The prevalence of malnutrition and its impact on all-cause mortality in patients with CHF were assessed using a meta-analysis.

**Methods:**

PubMed, Embase, the Cochrane Library, Web of Science, Medline, CBM, CNKI, WANFANG DATA, and VIP databases were searched to collect cross-sectional and cohort studies on malnutrition, and the prevalence and all-cause mortality of patients with CHF were determined. The time of retrieval was from the database establishment to May 2021. Two researchers independently performed screening of the literature, data extraction and assessed the risk of bias in the included studies. Then Stata 16.0 software was used for meta-analysis.

**Results:**

A total of 10 cross-sectional and 21 cohort studies were included, including 12537 patients with CHF. A meta-analysis demonstrated that the total prevalence of malnutrition in patients with heart failure was 46% (95% confidence interval [CI]: 0.43, 0.49). Compared to patients with non-malnutrition, malnutrition increased the risk of all-cause mortality in patients with CHF (hazard ratio = 2.15, 95% CI [1.89, 2.45], P < 0.05).

**Discussion:**

Current evidence suggests that the prevalence of malnutrition is high among patients with CHF. The risk of all-cause mortality in such patients can be increased by malnutrition. Therefore, the risk of malnutrition in patients with CHF should be considered to reduce the occurrence of adverse clinical outcomes.

## Introduction

Heart failure (HF) is an aggregate clinical syndrome, where various structural or functional diseases lead to ventricular filling and/or ejection dysfunction. The heart is unable to maintain sufficient cardiac output to fulfill the requirements of energy metabolism and adapt to the amount of venous return. Therefore, HF is the end stage of various cardiovascular diseases. HF is a commonly occurring disease, affecting 1%–2% of the population worldwide [1]. Malnutrition is a common complication in patients with CHF. Epidemiological evidence in patients with HF demonstrates that malnutrition mostly occurs simultaneously with chronic heart failure (CHF). The progressive decline of ejection function in patients with CHF leads to blood stasis in systemic and pulmonary circulation, resulting in slow gastrointestinal peristalsis, abnormal secretion of digestive enzymes, and a high metabolism caused by cytokines, further leading to dystrophic absorption [2]. Thus, in such a case, patients with HF will lose weight and will demonstrate symptoms such as fatigue, dyspnea, decreased daily activities, decreased muscle volume and weakness, cognitive impairment, and dysphagia. Therefore, the prognosis gradually worsens. Simultaneously, with the progress of the disease course, patients with CHF are often accompanied by depression and/or anxiety and other adverse psychological states, and the body is in a state of prolonged vigorous catabolism. Patients with CHF have the potential risk of malnutrition and even cachexia [3]. According to reports, the all-cause mortality rate of malnutrition within 12 months is 5.0%–30.0%, and the 1-year hospitalization rate is 18.9%–65.0% [4]. Some studies have demonstrated that malnutrition may assist in predicting the all-cause mortality, risk of cardiovascular (CV) events, and hospitalization owing to HF [5–9]. However, the prevalence and consequences of malnutrition are uncertain and controversial. Therefore, this systematic review and meta-analysis aimed to investigate the prevalence and prognosis of malnutrition in patients with HF.

## Methods

### Protocol and registration

The systematic review and meta-analysis were conducted based on the Preferred Reporting Items for Systematic and meta-analysis (PRISMA) protocols and the Meta-analysis Of Observational Studies in Epidemiology checklist. This protocol was registered with the International Platform of Registered Systematic Review and Meta-Analysis Protocols (INPLASY) on June 19, 2021 (Registration number INPLASY202160063).

### Eligibility criteria

Study type must be cross-sectional or cohort study (prospective or retrospective). The participants were patients with CHF. The study group had specific malnutrition assessment criteria, divided into malnutrition (risk) and non-malnutrition. The outcomes included morbidity or all-cause mortality. The exclusion criteria included repetitive reports, no related outcome index, important outcome data incomplete or missing, and when the original authors could not obtain the data. Moreover, literature review, systematic review or meta-analysis, and conference summary were excluded. The participants included HF patients with other conditions. The control group is a non-malnutrition group. Using the same cohort study, the literature with the longest follow-up period or the largest sample size was included.

### Search strategy

We searched PubMed, Embase, the Cochrane Library, Web of Science, CBM, CNKI, WANFANG DATA, and VIP databases to collect cross-sectional and cohort studies on the relationship between malnutrition and the prevalence and prognosis of patients with CHF. The retrieval time was from the establishment of the database to May 2021. Simultaneously, online databases and manual searches were used, and the references of the included literature were supplemented. The databases were searched with the following search terms using PICOS strategy by combining with AND, OR Boolean operators as heart failure OR cardiac failure OR HF AND malnutrition OR undernutrition OR nutrition disorder AND mortality OR death OR outcomes AND prevalence OR incidence. This meta-analysis was performed according to the PRISMA statement.

### Literature screening and data extraction

Two researchers independently performed literature screening, data extraction, and cross-checked it. Any difference was settled through negotiation after discussion with a third party. First, the titles of the articles were read. After excluding the irrelevant literature, the abstract and full text were further read to determine the literature that fulfilled the inclusion criteria. The extracted information included the first author, year of publication, type of study design, follow-up time, age of study participants, total sample size, type of heart failure, and adjustment factors.

### Assessment of methodological quality

Two related professional evaluators used the agency for healthcare research and quality (AHRQ) scale to evaluate the quality of the cross-sectional study. There were 11 items on the scale, with a total score of 11.0. A score of 8.0–11.0 indicated high quality, 4.0–7.0 medium quality, and < 4.0 indicated low quality [10]. Newcastle-Ottawa scale (NOS) was used to independently evaluate the risk of bias and cross-check the results. NOS scale is an effective method to evaluate the quality of systematic reviews of observational studies. The evaluation includes three aspects: the selection of research objects (four points), control of confounding factors in the study cohort (two points), and judgment of outcome events (three points). There are eight items on the scale, with a total score of 9.0. The score of 7.0–9.0 indicates high quality, while 4.0–6.0 indicates medium quality [11].

### Data analysis

Statistical analysis was used to extract the data of malnutrition prevalence and prognosis of patients with HF in each study. Stata 16.0 was used for meta-analysis. The pooled values were prevalence and adjusted hazard ratio (HR). HR was used to evaluate the effect of malnutrition on the risk of death. The heterogeneity of the study was evaluated using I^2^ and Q-test. If I^2^ < 50%, P-value of Q-test > 0.05, fixed-effect model was used for analysis; If I^2^ ≥ 50%, P-value of Q-test < 0.05, random effect model was used. If I^2^< 50%, P < 0.05, it shows that although the heterogeneity is highly significant, the impact is quite small. When judging the consistency of evidence, I^2^ is better than the Q test. Meta-regression and subgroup analysis were performed to determine the source of heterogeneity. Funnel plot, Begg test, and Egger test were used to evaluate the publication bias. The effect indicators of this study were malnutrition prevalence, HR, and 95% confidence interval (CI), with P < 0.05 as the difference was statistically significant.

## Results

### Selection of studies

**Fig 1:**
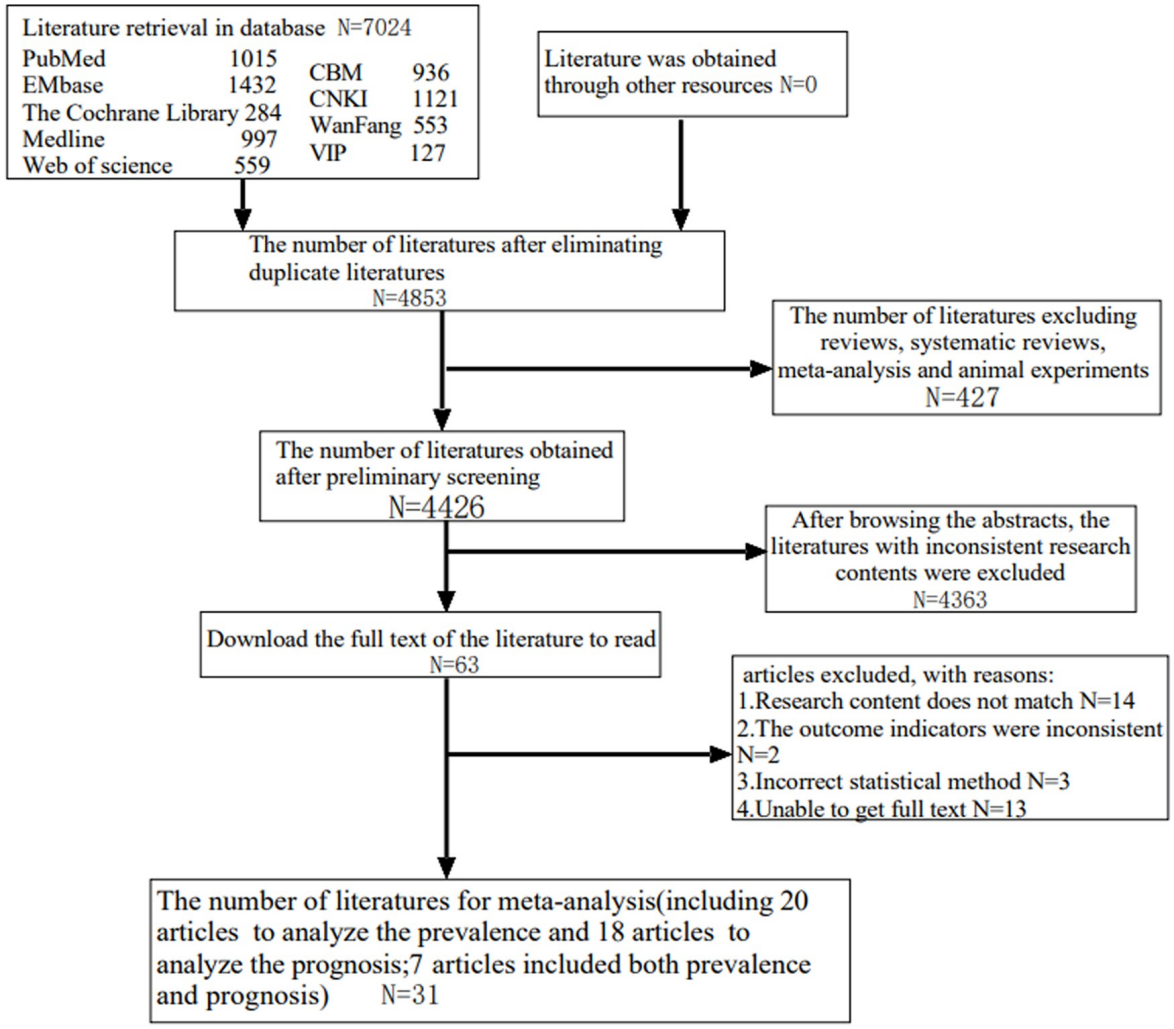
Flow chart of literature retrieval.

By searching the keywords, 7024 articles were obtained, including 2737 in Chinese and 4287 in English. According to the inclusion and exclusion criteria, 31 articles were included for meta-analysis (Fig 1).

### Description of included studies

The literature included 10 cross-sectional [12–21] and 21 cohort studies [5–9,22–37]. A total of 12537 patients with CHF were included, with seven included in both prevalence and prognosis studies [6–9,24,25,27]. Therefore, 20 articles (Table 1) were included in the prevalence study, and 18 (S1 Table) in the prognosis study. The included studies utilized different types of malnutrition screening and diagnostic tools, including nutritional risk screening (NRS2000); nutritional status control (CONUT); mini nutritional assessment (MNA); Geriatric Nutritional Risk Index (GNRI); nutritional risk index (NRI); prognostic nutritional index (PNI); anthropometry (body mass index, upper arm circumference, Skinfold thickness); and biomarkers (albumin, lymphocyte count). Only two studies [33,36], had all-cause mortality or rehospitalization as outcome indicators for HF, while the rest only had all-cause mortality. There were three articles [9,27,35] including two methods of malnutrition assessment for assessment of prognosis. The prevalence of malnutrition in patients with HF ranged from 37% to 56%. AHRQ was used to evaluate the quality of the cross-sectional study. The lowest score of literature was six points, and the highest was eight points, with an average of (7.7 ±0. 82). The quality of the cohort study was evaluated using the NOS literature quality assessment scale, of which four [5,23,24,28] were of medium quality, and the rest were of high quality.

**Table 1:** Basic information of included literatures: Prevalence of malnutrition.

Study	Research type	Types of heart failure	Age (years)	Sample size	Malnutrition	Prevalence	Evaluation criteria of malnutrition	Quality score
Pei 2014 [12]	Cross sectional study	HFrEF	59.5±10.7	48	20	0.42	Anthropometry and laboratory examination	8 points
Chen 2020 [13]	Cross sectional study	Unclear	Unclear	196	72	0.37	MNA	8 points
Wang 2018 [14]	Cross sectional study	Unclear	75.2±6.9	47	24	0.51	MNA	7 points
Zhou 2020 [15]	Cross sectional study	Unclear	Unclear	156	68	0.44	MNA	6 points
Hui 2021 [16]	Cross sectional study	Unclear	70.20±11.97	181	73	0.40	laboratory examination	8 points
Tevik 2015 [17]	Cross sectional study	HFrEF	78 (37–95)	131	56	0.43	NRS2002	9 points
Zhang 2018 [18]	Cross sectional study	Unclear	Unclear	300	145	0.48	NRS2002	8 points
Li 2020 [19]	Cross sectional study	Unclear	78.9±11.2	221	100	0.45	NRS2002	7 points
Lin 2015 [20]	Cross sectional study	HFrEF	61.8±15.0	100	49	0.49	GNRI	8 points
Li 2019 [21]	Cross sectional study	Unclear	71.4±2.5	200	78	0.39	MNA	8 points
Komorita 2020 [22]	Prospective cohort study	HFpEF	71.6±9.4	506	275	0.54	CONUT	7 stars
Yoshihisa 2018 [8]	Prospective cohort study	HFrEF	66.5	1274	570	0.45	GNRI	7 stars
Sze 2018 [9]	Prospective cohort study	HFrEF	73	1198	646	0.54	CONUT	7 stars
Wang 2020 [23]	Prospective cohort study	Unclear	74.6±6.6	241	113	0.47	GNRI	6 stars
La Rovere 2017 [24]	Prospective cohort study	HFrEF	61.3±11.0	466	251	0.54	CONUT	6 stars
Aziz 2011 [25]	Prospective cohort study	Unclear	72±14	1110	444	0.40	NRI	7 stars
Kinugasa 2013 [26]	Prospective cohort study	HFpEF	77±11	194	73	0.38	GNRI	7 stars
Nishi 2019 [6]	Prospective cohort study	HFpEF	78.5±7.2	110	49	0.45	GNRI	7 stars
Nakamura 2020 [7]	Prospective cohort study	HFrEF/HFpEF	87.2±4.9	213	112	0.53	GNRI	7 stars
Alataş 2020 [27]	Prospective cohort study	HFrEF/HFpEF	74.7±11.8	628	352	0.56	CONUT	7 stars

Unclear: The average age of samples or types of heart failure not mentioned in the literature; NRS2002: Nutritional Risk Screening; CONUT: COntrolling NUTritional Status Index; GNRI: Geriatric Nutritional Risk Index; MNA: Mini Nutritional Assessment; NRI: Nutritional Risk Index; HFrEF: Heart failure with decreased ejection fraction; HFpEF: Heart failure with preserved ejection fraction.

### Prevalence and prognosis of malnutrition

**Fig 2:**
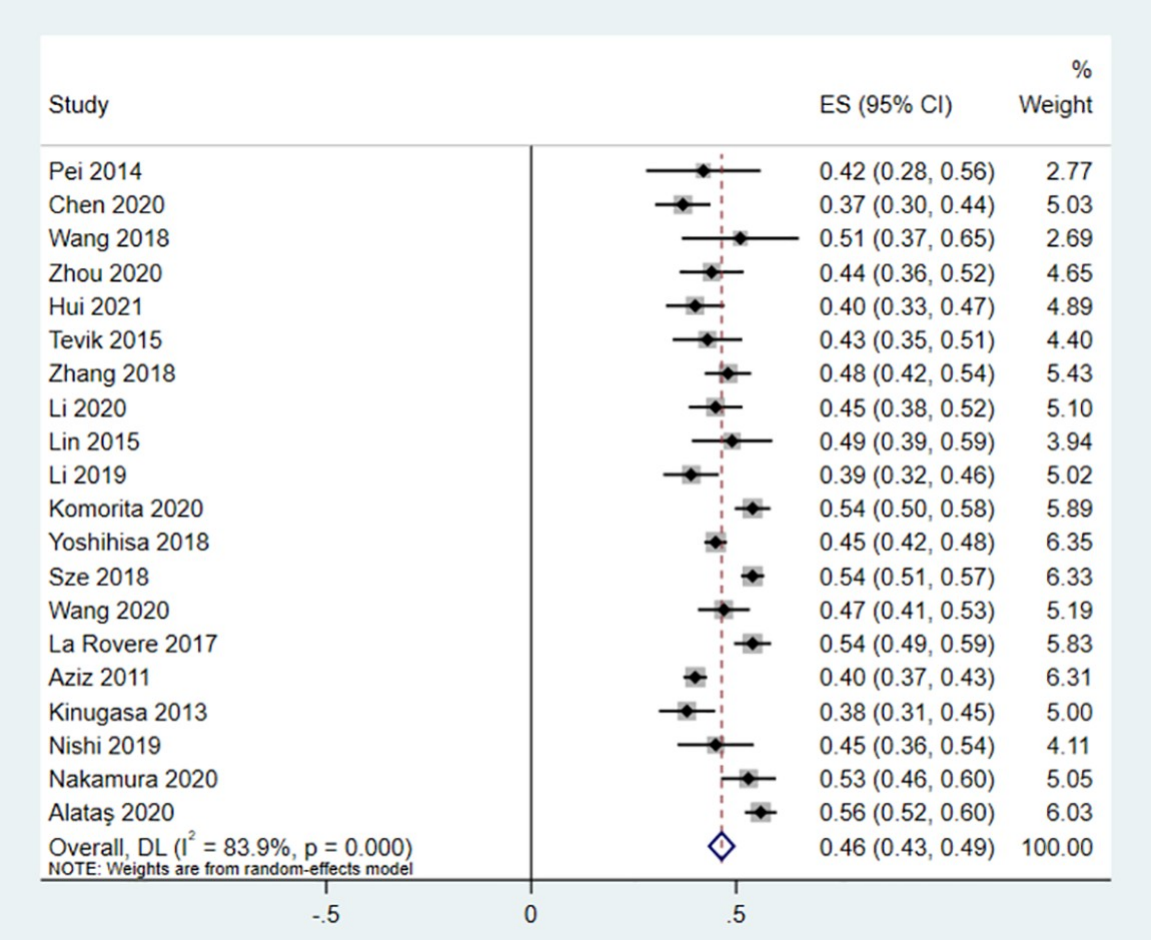
Forest plots of malnutrition prevalence in patients with chronic heart failure. ES: Effect Size CI: Confidence Interval DL: DerSimonian-Laird.

The prevalence study included 20 articles comprising 10 each of cross-sectional and cohort studies (baseline data describing the prevalence of malnutrition). A total of 7520 patients with HF, including 3570 patients with malnutrition, had a mean age of (73 ±9) years. There were 15 articles of high quality, and five of medium quality. The heterogeneity test of the included studies demonstrated that there was significant heterogeneity among the studies (I2 = 83.9%, P < 0.001); therefore, the random effect model was used. The results showed that the lowest prevalence of malnutrition was 37% (95% CI: 0.30–0.44), and the highest was 56% (95% CI: 0.52–0.60). The total prevalence of malnutrition was 46% (95% CI: 0.43 ~ 0.49), P < 0.001. These results were statistically significant (Fig 2).

**Fig 3:**
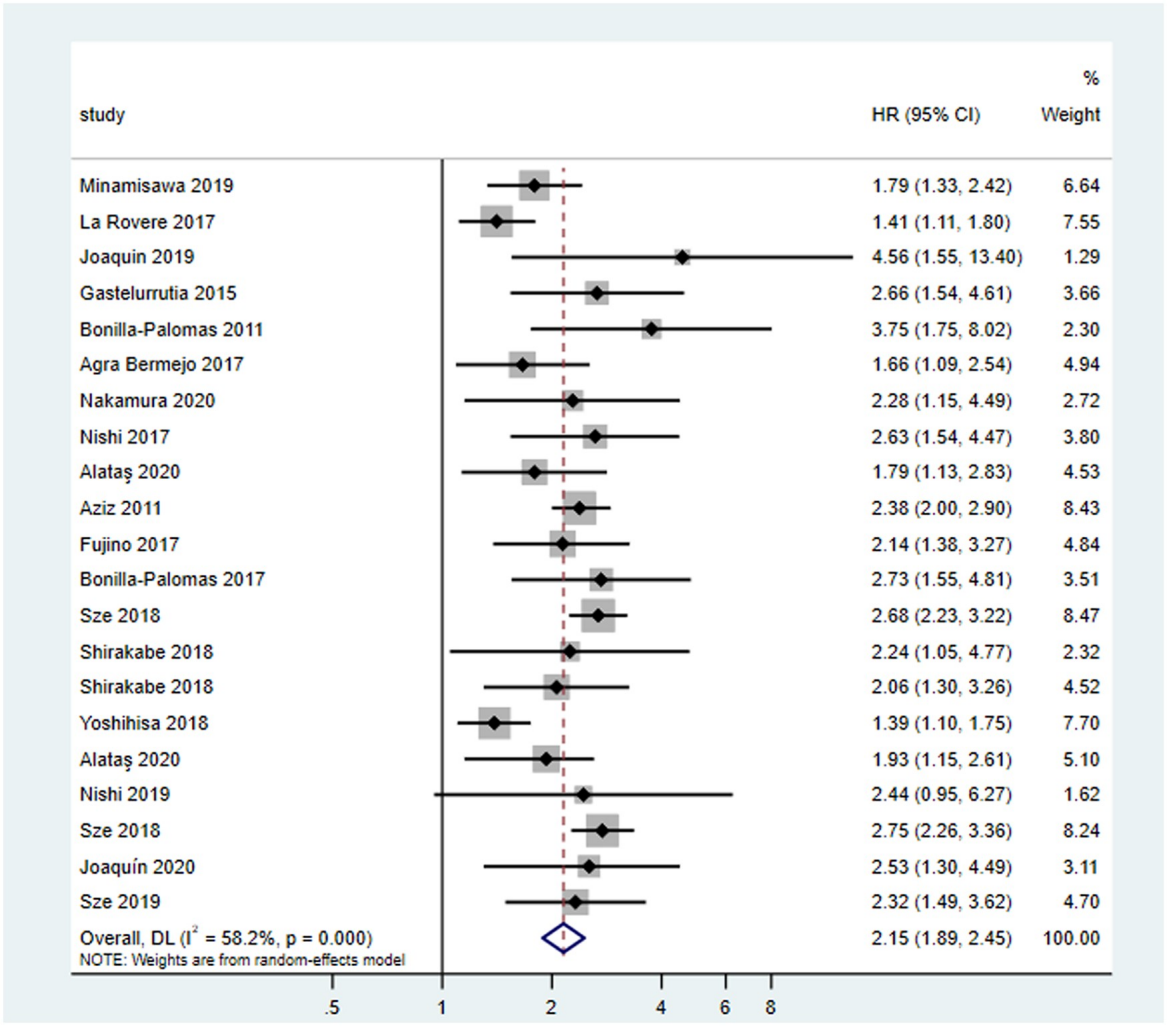
Forest plots of malnutrition prognosis in patients with chronic heart failure. HR: Hazard Ratio.

A total of 18 studies on prognosis were included, including one retrospective and 17 prospective cohort studies. A total of 10016 patients with HF were included in the study. The average age was (66.6 ±9.7) years. The average follow-up time was two years. There were three and 15 articles of medium and high quality, respectively. The assessment tools of malnutrition included GNRI, CONUT, MNA, anthropometry and laboratory examination, NRI, laboratory examination, and PNI. Eighteen articles were included in the heterogeneity test of the cohort study (I^2^ = 58.2% > 50%, and P < 0.1 of Q test), indicating that there was strong heterogeneity between the selected literature in this study (Fig 3). We can choose random effects for meta-analysis, and can continue to investigate the reasons for heterogeneity. Based on the data of this study, the source of highly suspected heterogeneity are malnutrition assessment criteria, and subgroup analysis will be conducted later. The random effects were selected for a total of 18 articles for meta-analysis, and the results were as follows: the results of meta-analysis given by random effects showed that the all-cause mortality of patients with malnutrition was almost twice than that of non-malnutrition patients with HF (HR = 2.15,95% CI [1.89,2.45]) (P < 0.05), and the results were statistically significant, indicating that malnutrition can significantly increase the risk of death.

### Meta-regression and subgroup analysis

Regarding prevalence studies, meta-analysis suggests that there is a high degree of heterogeneity among the included studies; therefore, meta-regression and subgroup analyses were conducted to study the source of heterogeneity. The results of regression analysis demonstrated that the malnutrition assessment tools used in the study (P = 0.02) might be the factors influencing the heterogeneity (S2 Table). The sub-group analysis was conducted by the research type, sample, nutritional assessment and screening tools, types of HF, and age group (S3 Table). The sub-group analysis revealed that the prevalence of malnutrition was comparable among elderly patients as compared to non-elderly patients, 48.4% (95% CI: 45.3–51.6) vs. 40.4% (95% confidence interval (CI): 38.1–42.7). The meta-analysis also revealed that the prevalence of malnutrition among patients with CHF was the highest with CONUT, GNRI, NRS2000 nutritional status screening, and assessment tools, while the prevalence of malnutrition was the lowest with MNA and others (anthropometry or laboratory examination). The subgroup analysis showed that there was almost no heterogeneity among the evaluation criteria of malnutrition. The prevalence rate among the subgroups did not fluctuate significantly and remained approximately 40%–50%.

Meta-analysis of this study on prognosis indicated that there was strong heterogeneity among the included studies; therefore, meta-regression and subgroup analyses were conducted to study the source of heterogeneity. The results of regression analysis showed that the malnutrition assessment tools used in the study (P = 0.04) may be the factors influencing the heterogeneity (S4 Table). Subgroup analysis showed that there was almost no heterogeneity among the evaluation criteria of malnutrition. It also revealed that the prognosis (all-cause mortality) of malnutrition among patients with CHF was the highest with MNA (HR = 3.0; 95% CI: 2.1–4.2) nutritional status screening and assessment tools, while the prognosis of malnutrition was the lowest with CONUT (HR = 1.6; 95% CI: 1.3–1.9] (S5 Table). There was no significant difference in all-cause mortality among subgroups. The overall all-cause mortality rate of malnutrition is about twice that of non-malnutrition.

### Sensitivity analysis

The results of sensitivity analysis demonstrated that there was no significant change in the prevalence and prognosis of malnutrition after excluding literature, indicating that the results of this meta-analysis were stable (S1 and S2 Figs).

### Publication bias

**Fig 4:**
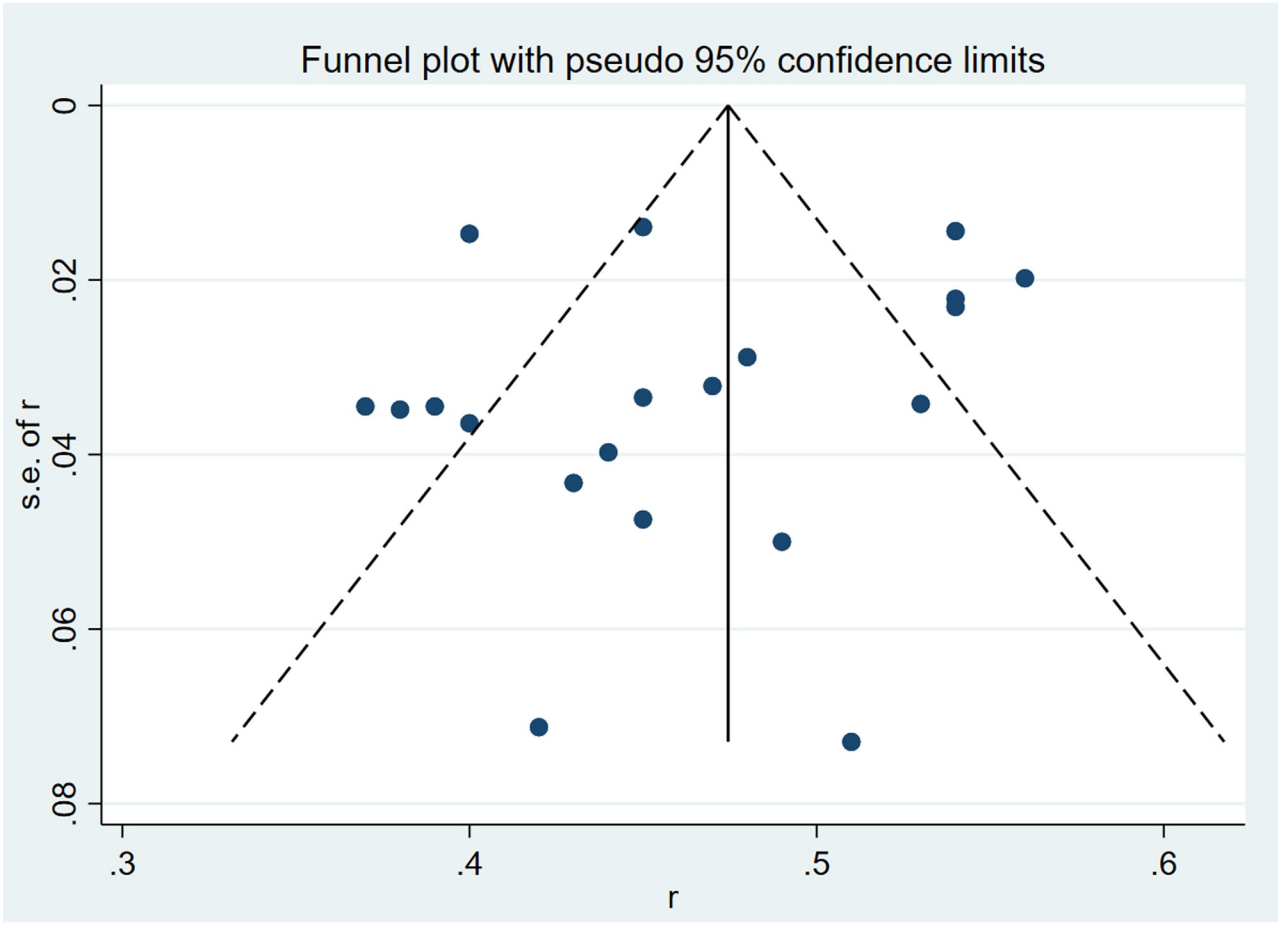
Funnel plot of literature publication bias on malnutrition prevalence in patients with chronic heart failure.

According to Begg, Egger test, and funnel plot, the included prevalence literature was tested for publication bias. Begg test showed that Kendall’s score was –26 (z = -0.84, P = 0.399) (S3 Fig), while the Egger test demonstrated that the difference was not statistically significant (t = –0.78, P = 0.444) (S4 Fig). The funnel plot showed that the scatter distribution of each study was symmetrical, without publication bias (Fig 4).

Similarly, according to the Begg, Egger test and funnel plot, a publication bias test was conducted for the included literature on the prognosis. The results of the Begg test showed that Kendall’s score was 50 (z = 1.51, P = 0.13) (S5 Fig). The results of the Egger test showed that there was no significant difference (t = 0.38, P = 0.708) (S6 Fig). The Funnel plot showed that the scatter distribution of each study was symmetrical, without publication bias (S7 Fig).

## Discussion

With the improvement of people’s living standards, the “three high” (hyperglycemia, hypertension, hyperlipemia) population is expanding annually, with the complications such as HF occurring commonly. Currently, HF is an increasingly critical health issue, requiring more attention. Malnutrition is common in patients with HF. In this meta-analysis, the prevalence rate was 46%. Amare [38] assessed the nutritional risk of 284 patients with HF, and the risk of malnutrition was observed as 77.8%. Yiqiu [39] screened 108 elderly patients with HF and found that the detection rate of malnutrition risk was 46.2%. Different national and international studies may be related to the majority of elderly people, more severe diseases, and different nutritional risk screening methods. This also shows that the risk of malnutrition in patients with HF is significantly increased than before. We should focus on the factors causing malnutrition in patients with HF and reduce their prevalence.

Currently, with the advent of an aging society, the number of patients with HF is expected to increase. These elderly patients are more prone to malnutrition, low activities of daily living, decreased muscle activity, and cognitive dysfunction. Malnutrition is caused by several factors, such as anorexia, malabsorption secondary to intestinal edema, high energy demand, and cytokine-induced hypermetabolism. Although many researchers have studied the mechanism of malnutrition in elderly patients with heart failure, they have not elucidated the molecular biological mechanism of malnutrition. We still need to conduct more in-depth research. To assess this situation, several assessment tools have been widely used. Because reasonable and accurate nutritional assessment is essential for the formulation of treatment strategies for elderly patients with heart failure. The Nutritional Index PNI (The Prognostic Nutritional Index), NRI (Nutritional Risk Index), GNRI (Geriatric Nutritional Risk Index), CONUT (COntrolling NUTritional Status Index), MNA (Mini Nutritional Assessment) only require simple and objective nutritional parameters (such as Plasma albumin level, plasma total cholesterol level, lymphocyte count and body weight) can be calculated. Owing to the simple calculation of the above nutritional indices, they are widely used to assess the nutritional status of elderly patients or patients with chronic diseases. MNA is difficult to be widely used to assess the nutritional status of all patients, while the calculation of nutritional index, viz., PNI, GNRI and COUNT, is relatively simple and easy to operate, and it is easier to be used to assess the nutritional status of elderly patients with heart failure.

Malnutrition leads to significant weight loss, considered to be the progress of cardiac cachexia as it is a state of catabolic consumption. Cardiogenic cachexia is one of the complications of chronic congestive HF, which is characterized by decreased body mass, changes in human components and disorder of multi system balance. Once developed, its prognosis is known to be devastating [40]. In addition, several studies have shown that the nutritional index PNI, GNRI and COUNT for the long-term prognosis of elderly patients with heart failure are significantly better than plasma albumin levels, total lymphocyte statistics, body mass index (BMI) and others.

The pathogenesis of malnutrition is complex and unclear. It is generally believed that many factors participate in the occurrence and development of malnutrition. It is well known that malnutrition is an independent prognostic factor leading to adverse clinical outcomes, including all-cause death or rehospitalization due to heart failure [41]. The literature included in this study evaluated the impact of malnutrition on the prognosis of patients with HF. The results showed that the risk of all-cause death in patients with malnutrition was twice as high as that in those without malnutrition. This result was consistent with the results of Wang *et al* [23]; however, there was a large difference between the results of Taro *et al* [42] and others, which may be related to the assessment tools and sample size of malnutrition.

Therefore, we consider that early nutritional intervention in patients with HF may provide better outcomes. Nutritional intervention improves the energy intake of patients with HF, enabling them to better cope with the catabolic/anabolic imbalance owing to neurohormonal activation and typical end-stage HF inflammation; thus, preventing its harmful effects [43]. Additionally, the nutritional intervention will help to optimize protein intake; thus, increase protein synthesis. This factor may help to maintain muscle energy to achieve better physical function exercise [44]. In the hospitalized patients with HF, hypoalbuminemia often exists. Research by Marijke et al. [43] showed that hypoalbuminemia can cause higher mortality. Similarly, nutritional intervention corrects hypoalbuminemia; thus, eliminating the negative impact of this condition on the patient’s prognosis. Numerous studies have shown that nutritional intervention can reduce the risk of all-cause death and readmission risk of HF deterioration in malnourished hospitalized patients with HF [45–47]. Therefore, evaluation of malnutrition in patients with HF is particularly important as different assessment methods may produce different nutrition-related risk results. Future studies should focus on better methods to estimate the nutritional status of patients with HF.

This meta-analysis analyzed the prevalence of malnutrition and its relationship with the all-cause mortality in patients with HF. However, the current systematic review and meta-analysis have some limitations. First, the sample size of some included studies was quite small, which might have influenced the results. Second, studies with information on malnutrition and mortality were excluded from the analysis. Third, some differences exist among different countries and populations, which might have affected some results. Additionally, all included studies were observational studies, which would have affected the accuracy of the study. Despite these limitations, malnutrition may be considered as one of the causes of mortality in patients with HF.

## Conclusion

The prevalence of malnutrition in patients with chronic HF is rising. Malnutrition can increase all-cause death in patients with HF. The evidence for the prevalence and prognosis of malnutrition in patients with HF was provided through this meta-analysis for prevention and early intervention as per the guidelines.

## Supporting information

S1 Table Basic information of included literatures: Prognosis of malnutrition. (DOCX)

S2 Table Meta regression analysis of malnutrition in patients with chronic heart failure. (DOCX)

S3 Table Prevalence of malnutrition among different subgroups. (DOCX)

S4 Table Meta regression analysis of malnutrition prognosis in patients with chronic heart failure. (DOCX)

S5 Table Prognosis of malnutrition among different subgroups. (DOCX)

S1 Fig Literature sensitivity analysis of malnutrition prevalence in patients with chronic heart failure. (DOCX)

S2 Fig Literature sensitivity analysis of malnutrition prognosis in patients with chronic heart failure. (DOCX)

S3 Fig Funnel plot generated by Begg test (prevalence). (DOCX)

S4 Fig Funnel plot generated by Egger test (prevalence). (DOCX)

S5 Fig Funnel plot generated by Begg test (prognosis). (DOCX)

S6 Fig Funnel plot generated by Egger test (prognosis). (DOCX)

S7 Fig Funnel plot of literature publication bias on malnutrition prognosis in patients with chronic heart failure. (DOCX)
