# Supplementary material for: The prevalence of malnutrition and its effects on the all-cause mortality among patients with heart failure: A systematic review and meta-analysis
Source: PLoS One. 2021 Oct 28;16(10):e0259300. doi: 10.1371/journal.pone.0259300 (PMC8553374; doi:10.1371/journal.pone.0259300)
Supplement: S1 Table — (DOCX) [file pone.0259300.s001.docx]

**S1 Table. Basic information of included literatures: prognosis of malnutrition**

| Study | Research type | Sample size | Types of heart failure | Age (years) | Evaluation criteria of  malnutrition | Follow up time | Adjust HR | Quality score | Outcome |
| --- | --- | --- | --- | --- | --- | --- | --- | --- | --- |
| Minamisawa 2019^5^ | Prospective cohort study | 1677 | Unclear | 73.4 (65.4, 80.0) | GNRI | 2.9 years | Yes | 6 stars | All cause death |
| La Rovere 2017^24^ | Prospective cohort study | 466 | HFrEF | 61.3±11.0 | CONUT | 12 months | Yes | 6 stars | All cause death |
| Joaquin 2019^28^ | Prospective cohort study | 151 | Unclear | 68.6 ± 10.9 | MNA | 2 years | Yes | 6 stars | All cause death |
| Gastelurrutia 2015^29^ | Prospective cohort study | 214 | HFrEF | 68.7 ± 11.4 | Anthropometry and laboratory examination | 2 years | No | 7 stars | All cause death |
| Bonilla-Palomas 2011^30^ | Prospective cohort study | 211 | Unclear | 73±10 | MNA | 22.1±11.6 months | Yes | 7 stars | All cause death |
| Agra Bermejo 2017^31^ | Retrospective cohort study | 145 | Unclear | 69,8 ± 11,0 | CONUT | 326 days | Yes | 7 stars | Hospitalization for heart failure |
| Nakamura 2020^7^ | Prospective cohort study | 213 | HFrEF/HFpEF | 87.2 ± 4.9 | GNRI | 540 days | Yes | 7 stars | All cause death |
| Nishi 2017^32^ | Prospective cohort study | 482 | HFrEF | 71.7 ± 13.6 | CONUT | 541.5 days | Yes | 7 stars | All cause death |
| Alataş 2020^27^ | Prospective cohort study | 628 | HFrEF/HFpEF | 74.7 ± 11.8 | CONUT | Unclear | Yes | 7 stars | All cause death |
| Aziz 2011^25^ | Prospective cohort study | 1110 | Unclear | 72±14 | NRI | 4 years | No | 7 stars | All cause death |
| Fujino 2017^33^ | Prospective cohort study | 432 | Unclear | 73 (64–82) | laboratory examination | 3 years | Yes | 8 stars | All cause death or  Hospitalization for heart failure |
| Bonilla-Palomas 2017^34^ | Prospective cohort study | 304 | Unclear | 74.6 ± 10.1 | MNA | 28 months | Yes | 7 stars | All cause death |
| Sze 2018^9^ | Prospective cohort study | 1198 | HFrEF | 73（63-79） | GNRI | 1 years | No | 7 stars | All cause death |
| Shirakabe 2018^35^ | Prospective cohort study | 458 | HFrEF | 76 (67–82) | CONUT | 365 days | Yes | 7 stars | All cause death |
| Shirakabe 2018^35^ | Prospective cohort study | 458 | HFrEF | 76 (67–82) | PNI | 365 days | Yes | 7 stars | All cause death |
| Yoshihisa 2018^8^ | Prospective cohort study | 710 | HFrEF | 66.5 | CONUT | 1146 days | Yes | 7 stars | All cause death |
| Alataş 2020^27^ | Prospective cohort study | 628 | HFrEF/HFpEF | 74.7 ± 11.8 | GNRI | Unclear | Yes | 7 stars | All cause death |
| Nishi 2019^6^ | Prospective cohort study | 110 | HFpEF | 78.5 ± 7.2 | GNRI | 1 years | Yes | 7 stars | All cause death |
| Sze 2018^9^ | Prospective cohort study | 1198 | HFrEF | 73（63-79） | PNI | 1 years | No | 7 stars | All cause death |
| Joaquín 2020^36^ | Prospective cohort study | 555 | HFmrEF | 69±11.5 | MNA | 23.8±6.6 months | Yes | 8 stars | All cause death or Hospitalization for heart failure |
| Sze 2019^37^ | Prospective cohort study | 952 | HFrEF/HFnEF | 80 (74–84) | GNRI | 1683 days | Yes | 8 stars | All cause death |

【NOTE】unclear: Follow up time or types of heart failure not mentioned in the literature; CONUT: COntrolling NUTritional Status Index; GNRI: Geriatric Nutritional Risk Index; MNA: Mini Nutritional Assessment ; NRI: Nutritional Risk Index; HFrEF: heart failure with decreased ejection fraction; HFpEF: heart failure with preserved ejection fraction
