# Supplementary material for: The prevalence of malnutrition and its effects on the all-cause mortality among patients with heart failure: A systematic review and meta-analysis
Source: PLoS One. 2021 Oct 28;16(10):e0259300. doi: 10.1371/journal.pone.0259300 (PMC8553374; doi:10.1371/journal.pone.0259300)
Supplement: S2 Table — (DOCX) [file pone.0259300.s002.docx]

**S2 Table. Meta regression analysis of malnutrition in patients with chronic heart failure**

|  | coefficient | P | 95% CI |
| --- | --- | --- | --- |
| Evaluation criteria of  malnutrition | 0.97 | 0.02 | (0.95，1.00) |
| sample size | 1.02 | 0.36 | (0.97，1.07) |
| Elderly | 1.01 | 0.64 | (0.95，1.09） |
| Types of heart failure | 1.02 | 0.28 | (0.98,1.05) |
