# Supplementary material for: The prevalence of malnutrition and its effects on the all-cause mortality among patients with heart failure: A systematic review and meta-analysis
Source: PLoS One. 2021 Oct 28;16(10):e0259300. doi: 10.1371/journal.pone.0259300 (PMC8553374; doi:10.1371/journal.pone.0259300)
Supplement: S3 Table — (DOCX) [file pone.0259300.s003.docx]

**S3 Table. Prevalence of malnutrition among different subgroups**

|  | Prevalence（%） | 95% CI | *I^2^* (%) | *Z* | P | Model |
| --- | --- | --- | --- | --- | --- | --- |
| All studies | 46.4 | (43.4, 49.5) | 83.9 | 30.00 | <0.001 | Random model |
| Research type |  |  |  |  |  |  |
| Cross sectional study | 43.2 | (40.5, 46.0) | 19.0 | 30.69 | <0.001 | Random model |
| Prospective cohort study | 48.8 | (44.5, 53.1) | 90.2 | 22.39 | <0.001 | Random model |
| Sample＞500 |  |  |  |  |  |  |
| Yes | 49.7 | (43.5, 55.9) | 94.7 | 15.65 | <0.001 | Random model |
| No | 45.0 | (41.9, 48.2) | 62.8 | 28.22 | <0.001 | Random model |
| Elderly |  |  |  |  |  |  |
| Yes | 48.4 | (45.3, 51.6) | 80.2 | 30.24 | <0.001 | Random model |
| No | 40.4 | (38.1, 42.7) | 0.0 | 34.66 | <0.001 | Random model |
| Evaluation criteria of  malnutrition |  |  |  |  |  |  |
| CONUT | 54.5 | (52.6,56.3) | 0.0 | 57.84 | <0.001 | Random model |
| GNRI | 45.9 | (42.2, 49.6) | 51.4 | 24.42 | <0.001 | Random model |
| MNA | 40.8 | (36.1,45.5) | 26.3 | 17.04 | <0.001 | Random model |
| NRS2002 | 46.0 | (42.1,49.8) | 0.0 | 23.57 | <0.001 | Random model |
| Others | 40.1 | (37.4,42.7) | 0.0 | 29.92 | <0.001 | Random model |
| Types of heart failure |  |  |  |  |  |  |
| HFrEF | 48.0 | (43.8,52.1) | 81.5 | 22.78 | <0.001 | Random model |
| Unclear | 42.7 | (39.9,45.5) | 44.2 | 29.82 | <0.001 | Random model |
| HFrEF/ /HFpEF | 55.2 | (51.9,58.6) | 0.0 | 32.23 | <0.001 | Random model |
