# Supplementary material for: The prevalence of malnutrition and its effects on the all-cause mortality among patients with heart failure: A systematic review and meta-analysis
Source: PLoS One. 2021 Oct 28;16(10):e0259300. doi: 10.1371/journal.pone.0259300 (PMC8553374; doi:10.1371/journal.pone.0259300)
Supplement: S4 Table — (DOCX) [file pone.0259300.s004.docx]

**S4 Table. Meta regression analysis of malnutrition prognosis in patients with chronic heart failure**

| project | coefficient | P | 95% CI |
| --- | --- | --- | --- |
| Evaluation criteria of  malnutrition | 1.10 | 0.04 | (1.00，1.22) |
| sample size | 0.97 | 0.87 | (0.75，1.27) |
| Elderly | 0.95 | 0.80 | (0.65，1.43） |
| Types of heart failure | 1.81 | 0.25 | (0.64,5.15) |
