# Supplementary material for: The prevalence of malnutrition and its effects on the all-cause mortality among patients with heart failure: A systematic review and meta-analysis
Source: PLoS One. 2021 Oct 28;16(10):e0259300. doi: 10.1371/journal.pone.0259300 (PMC8553374; doi:10.1371/journal.pone.0259300)
Supplement: S5 Table — (DOCX) [file pone.0259300.s005.docx]

**S5 Table. prognosis of malnutrition among different subgroups**

|  | Prognosis | 95% CI | *I^2^* (%) | *Z* | P | Model |
| --- | --- | --- | --- | --- | --- | --- |
| All studies | 2.152 | (1.887, 2.453) | 58.2 | 11.44 | <0.001 | Random model |
| Sample＞500 |  |  |  |  |  |  |
| Yes | 2.136 | (1.786, 2.553) | 71.6 | 8.33 | <0.001 | Random model |
| No | 2.159 | (1.777, 2.622) | 36.3 | 7.75 | <0.001 | Random model |
| Elderly |  |  |  |  |  |  |
| Yes | 2.093 | (1.805, 2.428) | 61.2 | 9.76 | <0.001 | Random model |
| No | 2.448 | (2.057, 2.912) | 0.0 | 10.10 | <0.001 | Random model |
| Evaluation criteria of  malnutrition |  |  |  |  |  |  |
| CONUT | 1.599 | (1.345, 1.901) | 24.3 | 5.31 | <0.001 | Random model |
| GNRI | 2.270 | (1.926, 2.676) | 17.2 | 9.77 | <0.001 | Random model |
| MNA | 3.003 | (2.122, 4.248) | 0.0 | 6.21 | <0.001 | Random model |
| PNI | 2.572 | (2.025, 3.266) | 21.8 | 7.75 | <0.001 | Random model |
| Others | 2.367 | (2.011 ,2.786) | 0.0 | 10.37 | <0.001 | Random model |
| Types of heart failure |  |  |  |  |  |  |
| HFrEF | 2.152 | (1.709,2.710) | 76.4 | 6.52 | <0.001 | Random model |
| Unclear | 2.212 | (1.843,2.655) | 28.7 | 8.52 | <0.001 | Random model |
| HFrEF /HFpEF | 2.033 | (1.606,2.575) | 0.0 | 5.89 | <0.001 | Random model |
