# Supplementary figures and images for: The prevalence of malnutrition and its effects on the all-cause mortality among patients with heart failure: A systematic review and meta-analysis
Source: PLoS One. 2021 Oct 28;16(10):e0259300. doi: 10.1371/journal.pone.0259300 (PMC8553374; doi:10.1371/journal.pone.0259300)

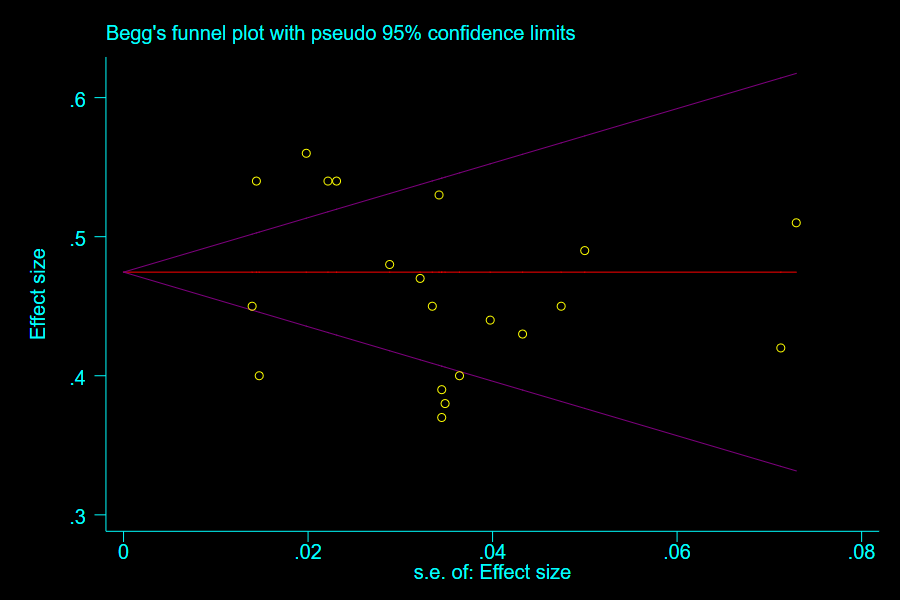


**S3 Figure. Funnel plot generated by Begg test (prevalence)**

Supplement: S3 Fig — (DOCX) [file pone.0259300.s008.docx]

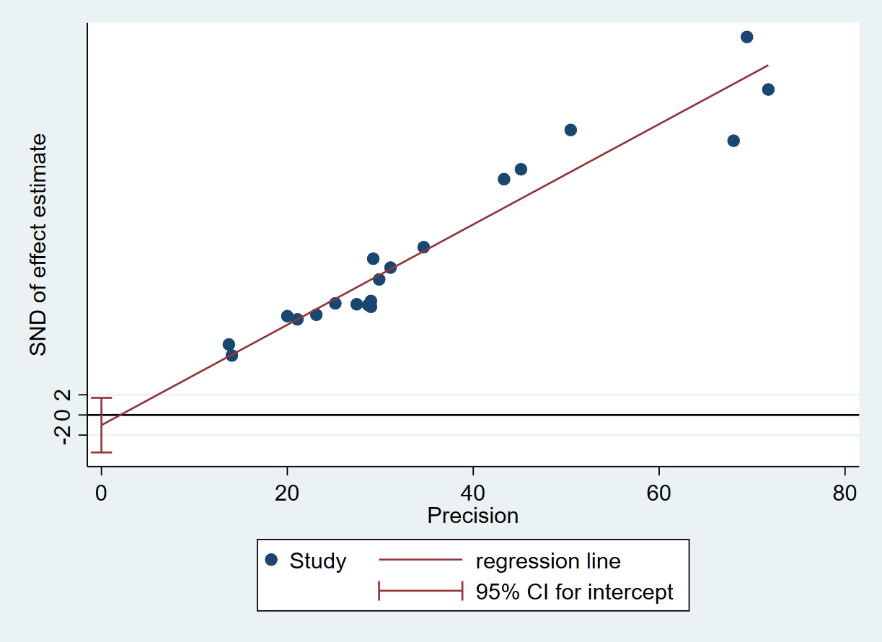


**S4 Figure. Funnel plot generated by Egger test (prevalence)**

Supplement: S4 Fig — (DOCX) [file pone.0259300.s009.docx]

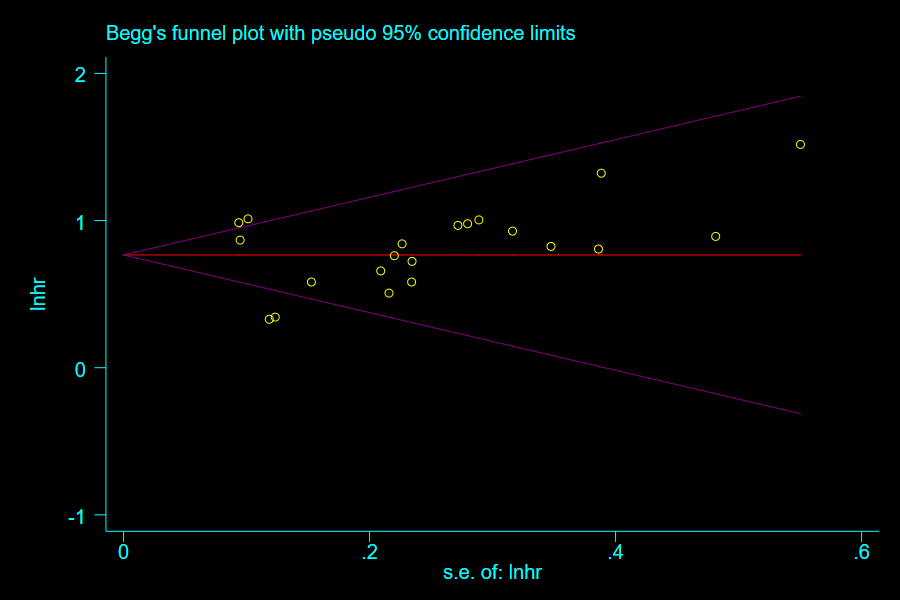


**S5 Figure. Funnel plot generated by Begg test (prognosis)**

Supplement: S5 Fig — (DOCX) [file pone.0259300.s010.docx]

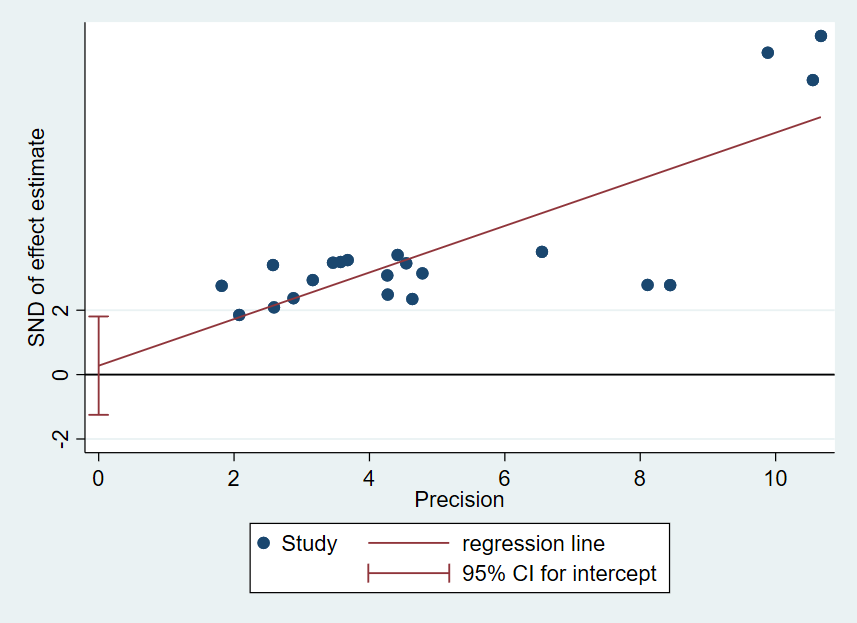


**S6 Figure. Funnel plot generated by Egger test（prognosis）**

Supplement: S6 Fig — (DOCX) [file pone.0259300.s011.docx]
