# Supplementary material for: The prevalence of malnutrition and its effects on the all-cause mortality among patients with heart failure: A systematic review and meta-analysis
Source: PLoS One. 2021 Oct 28;16(10):e0259300. doi: 10.1371/journal.pone.0259300 (PMC8553374; doi:10.1371/journal.pone.0259300)
Supplement: S7 Fig — (DOCX) [file pone.0259300.s012.docx]

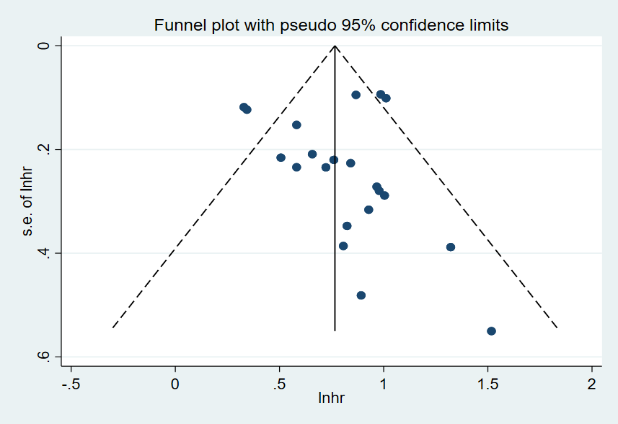


**S7 Figure. Funnel plot of literature publication bias on malnutrition prognosis in patients with chronic heart failure**
